# *Metarhizium pingshaense* infection reverses insecticide resistance in *Anopheles gambiae* sensu lato by altering energy reserves and gene expression

**DOI:** 10.1186/s13071-025-07132-z

**Published:** 2025-11-28

**Authors:** Doubé Lucien Lamy, Francesco Baldini, Mafalda Viana, Iván Casas Gómez-Uribarri, Duangkamon Loesbanluechai, Erin S. Johnston, Meshach Lee, Najat Feruzi Kahamba, Edounou Jacques Gnambani, Issiaka Saré, Souro Abel Millogo, Moussa Namountougou, Abdoulaye Diabaté, Etienne Bilgo

**Affiliations:** 1 Institut de Recherche en Sciences de la Santé, Direction Régionale de l’Ouest, 01 BP 545, Bobo-Dioulasso, Burkina Faso; 2 Centre MURAZ/Institut National de Santé Publique (INSP), Bobo-Dioulasso, Burkina Faso; 3 Unité de Formation et de Recherche en Sciences de la vie et de la Terre, Université Nazi BONI, Bobo-Dioulasso, Burkina Faso; 4 Laboratoire d’Entomologie Fondamentale et Appliquée (LEFA), Université Joseph Ki-Zerbo, 03 BP 7021, Ouagadougou, Burkina Faso; 5 School of Biodiversity One Health and Veterinary Medicine, University of Glasgow, Glasgow, G12 8QQ UK; 6 Ecological Sciences Department, Ifakara Health Institute, Environmental Health, Morogoro, Tanzania

**Keywords:** Malaria, *Metharizium pingshaense*, Insecticide, Reverting resistance, Fitness, Immune genes, Anopheles gambiae s.l.

## Abstract

**Background:**

Entomopathogenic fungi like *Metarhizium* are emerging as effective biopesticides against malaria vectors. They reduce mosquito survival, fecundity, and flight ability, and reverse insecticide susceptibility in resistant *Anopheles gambiae* sensu lato strains. To elucidate the unclear underlying mechanisms, this study investigates the effects of fungal infections and insecticide exposure on the mosquito’s energy reserves and the expression of key metabolic and immune genes.

**Methods:**

Three mosquito types: (i) pyrethroid-resistant *An. gambiae* sensu lato and two laboratory colonies: (ii) pyrethroid-resistant *An. coluzzii* VKPER and (iii) insecticide-susceptible *An. gambiae* sensu stricto Kisumu were used. They were infected with *Metarhizium pingshaense* S10 strain at a concentration of 10⁷ spores/mL (treatment groups) and with solvent only (0.05% Tween^®^ 80; control groups). Live mosquitoes were collected on days 0, 4, and 8 post-infection. They were used to quantify glucose, glycogen, and lipid via Van Handel’s protocol and to assess insecticide resistance. For resistance testing, mosquitoes underwent a standard WHO insecticide susceptibility test using deltamethrin (0.05%) or a control. Survival was measured 1 h after exposure, and surviving mosquitoes were analyzed by RT-qPCR for the expression of *defensin* and *CYP6P3*, *CYP6Z1*, and *GSTe2*.

**Results:**

Susceptible *An. gambiae* Kisumu were eliminated by deltamethrin, while resistant *An. coluzzii* VKPER and wild *An. gambiae* s.l. mosquitoes survived. However, deltamethrin exposure following *Metarhizium* infection significantly reduced survival in these resistant strains compared to the controls. This also resulted in reduced expression levels of *defensin*, *GSTe2*, and *CYP6Z1* compared to deltamethrin exposure alone, but no difference was found in the expression levels of *CYP6P3*. These results collectively indicate that *Metarhizium* infection reduces mosquito survival by impairing their energetic reserves and ability to sustain vital physiological processes, including immune function and metabolic homeostasis.

**Conclusions:**

We demonstrate that *Metarhizium* infection reverses insecticide resistance in *An. gambiae* s.l. by depleting energy reserves and suppressing the expression of detoxification genes. This mechanistic insight is crucial for optimizing the future integration of *Metarhizium* alongside conventional insecticides for malaria vector control.

**Graphical Abstract:**

**Figure:**
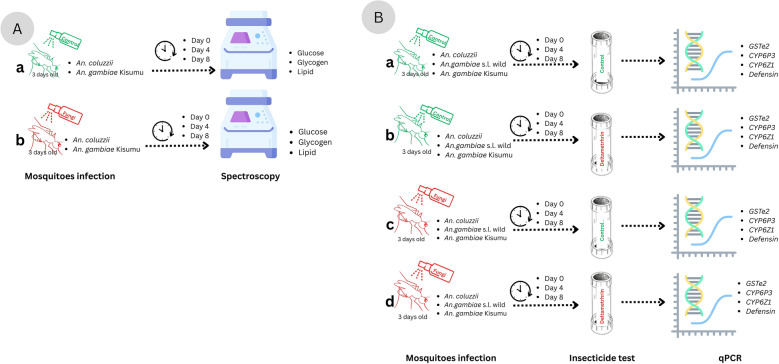

**Supplementary Information:**

The online version contains supplementary material available at 10.1186/s13071-025-07132-z.

## Background

In the early twenty-first century, malaria gained recognition worldwide as a priority global health issue, prompting intensive control efforts. These efforts have led to substantial progress in reducing *Plasmodium falciparum* infections in humans, with an estimated 50% decline in Africa and a 40% reduction in clinical malaria incidence between 2000 and 2015 [1, 2]. Vector control measures, which remain a pivotal public health strategy for malaria control, have been credited for this success [3], particularly two insecticidal interventions: long-lasting insecticide-treated nets (LLINs), which are bed nets treated with synthetic pyrethroid, and indoor residual spraying (IRS) [1, 3]. However, the intensive use of these tools has led to insecticide resistance to pyrethroids, the only class of insecticides approved for use in bednets, posing a significant threat to the progress of malaria control [4–6]. In addition to insecticide resistance, other factors such as limited access to healthcare, changing environmental conditions, and parasite resistance to antimalarial drugs also contribute to the persistent burden of malaria. As a result, the disease continues to cause hundreds of millions of cases and hundreds of thousands of deaths each year [7]. According to the latest World Malaria Report [8], malaria caused 246 million cases and claimed 569,000 lives in Africa in 2023 [9]. This underscores the need to develop innovative tools for sustainable malaria control, not only as alternatives or complements to existing strategies [6, 10], but also to understand the mechanisms of action for better integration.

Two main insecticide resistance mechanisms have been described in African malaria vectors: (i) target-site resistance, which prevents insecticide binding by altering insecticide target sites [11–14], and (ii) metabolic resistance, characterized by the overexpression of enzyme systems that enhance the detoxification or sequestration of insecticides [15–17]. This latter mechanism involves the action of cytochrome P450-dependent monooxygenases (CYPs), carboxylesterase, and glutathione S-transferases (GSTs) [18, 19]. Insecticide synergists, which are non-toxic chemicals added to insecticides to increase their lethality, or more generally, their effectiveness, have been proposed as a management strategy for metabolic insecticide resistance [20]. For example, piperonyl butoxide (PBO) is currently used in LLINs to improve the efficacy of pyrethroid-treated bed nets by inhibiting mosquito detoxification enzymes, ultimately increasing insecticide effectiveness [21, 22]. In malaria-endemic regions with insecticide resistance, PBO-treated nets can reduce malaria cases by more than 33% compared with conventional LLINs, making them a key control strategy [23–25]. However, PBO-LLINs may not have a significant effect in areas where metabolic resistance is not prevalent [26] or if mosquitoes develop high levels of P450 enzyme activity [23, 27].

Biopesticides such as the fungus *Metarhizium* offer a promising alternative to synthetic chemical insecticides for the control of malaria vectors. These agents are safer for the environment and can help mitigate the growing issue of insecticide resistance among mosquitoes [28–30]. Unlike synthetic chemical insecticides, biopesticides are generally host-specific and safer for non-target organisms, thereby reducing environmental impact [31]. *Metarhizium* infection has been shown to affect mosquito fitness [32, 33]. For example, *Metarhizium anisopliae* can disrupt the mosquito life cycle by shortening its lifespan and reducing fecundity [34], ultimately affecting population dynamics. Previous studies have also shown that *Metarhizium* can complement pyrethroids and potentially mitigate their resistance in mosquito populations [35–38]. Recently, we investigated the effects of combining these fungi and deltamethrin on insecticide-resistant mosquitoes and found that exposing *Metarhizium*-infected mosquitoes to deltamethrin significantly increased mortality compared to that in the non-infected group, suggesting that fungal infection can reverse insecticide resistance [35]. However, the mechanism has not yet been elucidated.

Mosquito fitness is closely related to energy levels and immunity. Mosquitoes emerge with a limited energy reserve, known as teneral energy, and quickly seek additional sources, such as nectar and blood meals, to survive and reproduce [39, 39–42]. Excess energy is stored as proteins, glycogen, and lipids, which support essential activities such as reproduction and flight [43–45]. For example, glycogen primarily fuels short-term flights, whereas lipids sustain longer flights [46]. Here, we investigated the mechanisms underlying insecticide resistance reversal by exploring changes in metabolic insecticide resistance, immune gene expression, and energy reserves in fungus-infected mosquitoes.

## Methods

### Mosquito strain

For the bioassays, we used three mosquito types: (i) pyrethroid-resistant *An. coluzzii* VKPER strain; (ii) insecticide-susceptible *An. gambiae* s.s. Kisumu, and (iii) the wild-caught strain *An. gambiae* s.l. The laboratory pyrethroid-resistant *An. coluzzii* VKPER colony was established from mosquitoes collected at Valley du Kou in Burkina Faso and selected continuously to maintain the *kdr* gene fixed [13, 47]. All strains were maintained and made available at the Institut de Recherche en Science de la Santé (IRSS), Burkina Faso. Larvae of *An. gambiae* s.l. were collected in October and November 2023 from several natural breeding sites in Soumousso and Valley du Kou and brought to the insectary at the IRSS in Bobo Dioulasso, where they were reared to adulthood. The larvae were fed with Tetramin fish food daily. Upon emergence, the adults were morphologically identified using an identification key, and only *An. gambiae* s.l. were maintained. All mosquito strains were maintained in the insectary under the same optimal conditions at 27 ± 2° C, 80 ± 10% relative humidity, and a photoperiod of 12:12 (L:D) at IRSS. Only 3–5-day-old, non-blood-fed females were used for the bioassays.

### Mosquito infection

The *Metarhizium pingshaense* S10 strain used in all infections in this study was previously isolated from wild *An. gambiae* s.l. collected in an inhabited house in Soumousso (11°04ʹN, 4°03ʹW), Burkina Faso [28]. Here, mosquito infections were carried out using spore suspension of 14–30 day culture suspended in 0.05% Tween (Polysorbate Tween^®^ 80) at a final concentration of 10^7 ^spores/ml. Conidial concentration was determined using a Neubauer hemocytometer. The fungal suspension was mixed vigorously by using a vortex mixer, and then 0.25 ml of the suspension was sprayed on 80 mosquitoes in the treatment group, whereas the control group was sprayed with 0.25 ml of conidia-free 0.05% Tween solution using an atomizer. Mosquitoes were first anesthetized at -20 °C for 15 s and then sprayed with the fungal solution or the same volume of 0.05% Tween 80 solution for the control before their recovery. After the infection, mosquitoes were allowed to wake up in cups, transferred to small cages, and provided with 5% glucose solution.

### Sampling and experimental procedures

**Fig. 1:**
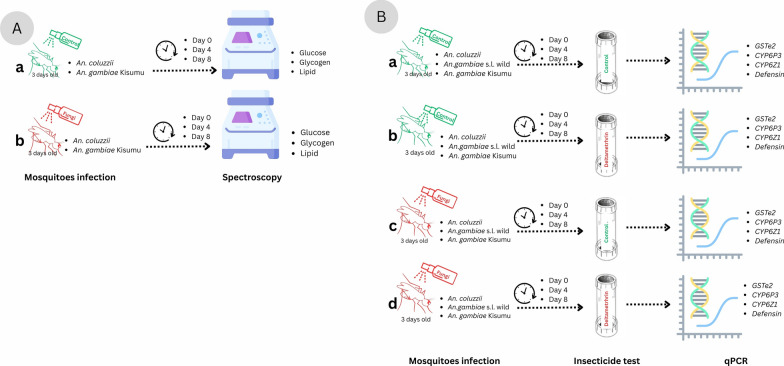
Schematic representation of the experimental design. **A**: illustrates energy quantification experiment; **a** uninfected control, **b** *Metarhizium* control group. **B**: illustrates gene expression experiment; **a** control, **b** deltamethrin only, **c** *Metarhizium* only, and **d** combination of *Metarhizium* and deltamethrin

For each of the three replicates, 15 mosquitoes were randomly selected from each treatment group (*Metarhizium*-infected and control) at 1 h, 4 days, and 8 days post-infection. Mosquitoes were then briefly anesthetized at − 20 °C for 5 min, transferred to dry, labeled Falcon tubes, and immediately stored at − 80 °C for subsequent energy reserve quantification assays. A sampling protocol was conducted for *An. coluzzii* and *An. gambiae* Kisumu (Fig. 1A) but not wild *An. gambiae* s.l.

In addition to *Metarhizium* infection, mosquitoes used for gene expression quantification were also exposed to deltamethrin at 1 h, 4 days, and 8 days post-infection using the WHO insecticide susceptibility tests. This involved exposing 20–25 *Metarhizium*-infected mosquitoes to deltamethrin or control for 1 h duration. The mosquitoes were then transferred to insecticide-free observation tubes for an additional hour [48], after which mortality was recorded. Surviving mosquitoes were then pooled into groups of five and placed into 1.5 ml tubes containing 1 ml of RNAlater for subsequent gene expression analysis. This timeframe was chosen to preserve RNA integrity for the subsequent qPCR analysis of gene expression responses. The tubes were stored at 4 °C for 24 h to allow RNAlater to penetrate the tissues before being stored at -80 °C until RNA extraction (Fig. 1B). In both experiments, samples collected at 1 h (day 0) were used as the baseline for metabolite and gene expression quantification.

### Genes expression

#### RNA extraction

Total RNA was extracted from 12 pools of five mosquitoes per treatment group using the PureLinkTM RNA Mini Kit (Invitrogen) following the manufacturer’s instructions. RNA concentration was measured using a Tecan Infinite PRO fluorescence microplate reader. Samples with RNA concentration exceeding 100 ng/µl were diluted to 100 ng/µl before using them for reverse transcription.

#### Complementary DNA synthesis

For cDNA synthesis, a reverse transcription reaction was performed with 2 μl of buffer, 0.8 μl of dNTP mix, and 2 μl of random primers. Then, 1 μl of reverse transcriptase enzyme and 1 μl of RNase inhibitor were added. The volume was adjusted to 10 μl with nuclease-free water. An RNA sample of 10 μl was then added, resulting in a final reaction volume of 20 μl. The thermal protocol included primer annealing at 25 °C for 10 s, DNA polymerization at 37 °C for 2 min, and reverse transcriptase deactivation at 85 °C for 5 s, with a final hold at 4 °C. The resulting cDNA was diluted 1:10 before use in qPCR assays.

#### qPCR assays

The expression levels of four genes, *CYP6P3*, *CYP6Z1*, *GSTe2,* associated with insecticide metabolic resistance, and *defensin* associated with mosquito immune defense against pathogens, including bacteria and fungi, have been quantified [16]. The housekeeping gene encoding ribosomal protein S7 (RPS7) [16] was measured and used as a reference for overall gene expression. The qPCR reactions were run in volumes of 15 μl, containing 5 μl of template nucleic acid extract and 10 μl of master mix of specific primers. Primers were supplied by Integrated DNA Technologies, Inc. (1710 Commercial Park, Coralville, IA 52241, USA). All reactions were performed on Applied Biosystems, *ViiA™ 7 Real-Time PCR System with 96-Well Block*, Thermo Fisher Scientific, in MicroAmp^®^ Fast 96-Well Reaction Plate (0.1 ml) from Life Technologies Corporation^™^. The thermal protocol started with an initial denaturation at 95 °C for 1 min, followed by 40 cycles of 95 °C for 5 s, 60 °C for 10 s, and 72 °C for 30 s. A final extension at 65 °C for 30 s completed product synthesis. The procedure ended with a melt curve analysis: 95 °C for 30 s, 65 °C for 30 s, and 95 °C for 30 s, to confirm amplification specificity and result accuracy. The primers used in this study are listed in Table 1. These primers were designed specifically to target the conserved regions of each gene across both species. The corresponding AGAP gene identifiers for each primer pair are listed in Table 2.

**Table 1:** Primer sequences of target genes in *Anopheles gambiae* and *Anopheles coluzzii*

Gene	Code	Forward	Reverse
S7	AGAP009613	GAGTCGGAGGAGGAAAAGGC	CAACAGTGGGCGACAGTTTT
CYP6P3	AGAP002865	CTGTCTGTACGAGCTGGCAA	GTCGTACGTCACCTCTCCAC
CYP6Z1	AGAP008219	GCGGCCAATGTGTTTCTGTT	GCCTCAGCATTGTGCGTAAG
GSTe2	AGAP009194	CCAAAGCATTGGGCTTGGAG	AGCACCGGGATCGTATGTTG
Defensin	AGAP007200	AACATTCACTGTCGCGGGTA	CTTTCATCGCTGCCGGTTTG

**Table 2:** Pairwise comparison of mortality rate in wild *An. gambiae* s.l. and *An. coluzzii* after exposure to either control, deltamethrin, fungi, or mix (deltamethrin & fungi together)

Comparisons	Odds ratio	SE	*p*-value
Control/deltamethrin	0.008	0.008	< 10^–3^
Control/*metarhizium*	0.046	0.047	0.006
Control/mix	0.001	0.001	< 10^–3^
Deltamethrin/*metarhizium*	5.418	1.360	< 10^–3^
Deltamethrin/mix	0.148	0.018	< 10^–3^
*Metarhizium*/mix	0.027	0.006	< 10^–3^

### Energy reserves quantification

#### Extraction of lipid, glycogen, and sugar from mosquito pools

Energy reserves were quantified in 18 pools of five mosquitoes per treatment group according to the Van Handel protocol [49–51]. This protocol requires the separation of lipids, glucose, and glycogen. Pools of mosquitoes were homogenized in 200 µl of sodium sulfate using plastic pestles until no identifiable parts remained. A 1200 µl solution of chloroform/methanol (1:1) was then added to the homogenate and mixed. The homogenate was centrifuged at 3000*g* for 1 min. The supernatant containing lipids and glucose was transferred to a clean centrifuge tube, and the pellet was retained in the tube for glycogen analysis. Distilled water (600 µl) was added to the supernatant and mixed before centrifugation at 3000*g* for 1 min. The top phase was used for glucose quantification, whereas the bottom phase was used for lipid analysis. For heating purposes, both phases were transferred to glass tubes marked at the 5 mL level.

#### Glucose analysis

Glucose extracts were heated at 110 °C to evaporate the water/methanol solvents down to 200 µl before adding anthrone up to the 5 mL level mark. The solution was mixed, heated for 17 min at 110 °C, and removed from the heating block for cooling. The absorbance of the resulting solution was measured at 625 nm in duplicate. The glucose concentration was determined by comparing the absorbance to that of a standard made from 100 mg/100 ml of anhydrous glucose in deionized water.

#### Glycogen analysis

Anthrone was added to the pellet at the 5 ml level, then mixed and vortexed. The homogenate was heated to 110 °C for 17 min. The absorbance of the resulting solution was measured at 625 nm in duplicate. The glycogen concentration was obtained by comparing the absorbance to a standard consisting of 100 mg per 100 ml of anhydrous glucose in deionized water.

#### Lipids analysis

Lipid extracts were heated at 110 °C to evaporate the chloroform completely. Once dried, 200 µl of sulfuric acid was added, and the solution was heated at the same temperature for 10 min. Vanillin was then added to the 5 ml mark and mixed. The tube was then removed from the heating block and allowed to cool to room temperature. The absorbance of the resulting solution was measured at 625 nm in duplicate. The lipid concentration was determined by comparing the absorbance with that of the vegetable oil.

### Statistical analysis

The susceptibility of infected mosquitoes to deltamethrin was analyzed using a binomial Generalized Linear Model (GLM). The dependent variable was the proportion of mosquitoes dead within 1 h of exposure and treatment, with mosquito strain and infection duration included as explanatory variables. For this analysis, we considered the inclusion of replicates as a random effect and interaction term among explanatory variables, but they were not supported, and hence, were not included in the final models.

Energy reserves and gene expression were analyzed using Linear Mixed Models (LMM). The initial models included treatment, infection duration, mosquito strain, and the interaction between treatment and infection duration as explanatory variables, as well as random replicates. Because of repeated amplification failure of *GSTe2* in wild *An. gambiae* s.l., the data from this line were insufficient for analysis and were removed from the final model. Mosquito survival analysis was performed using the Cox proportional hazards (Coxph) model with treatment and mosquito strain as fixed effects and replicates as a random effect. All models were fitted to R (v4.3.3) using the package lme4 (v1.1.35.2) for LMM, and survival (v3.5.8) for Coxph. Standard tests were conducted to verify the model assumptions using the DHARMa package (v0.4.6). Inferences were made using likelihood ratio (LRT) tests.

## Results

### *Metarhizium* infection reverses insecticide resistance

**Fig. 2:**
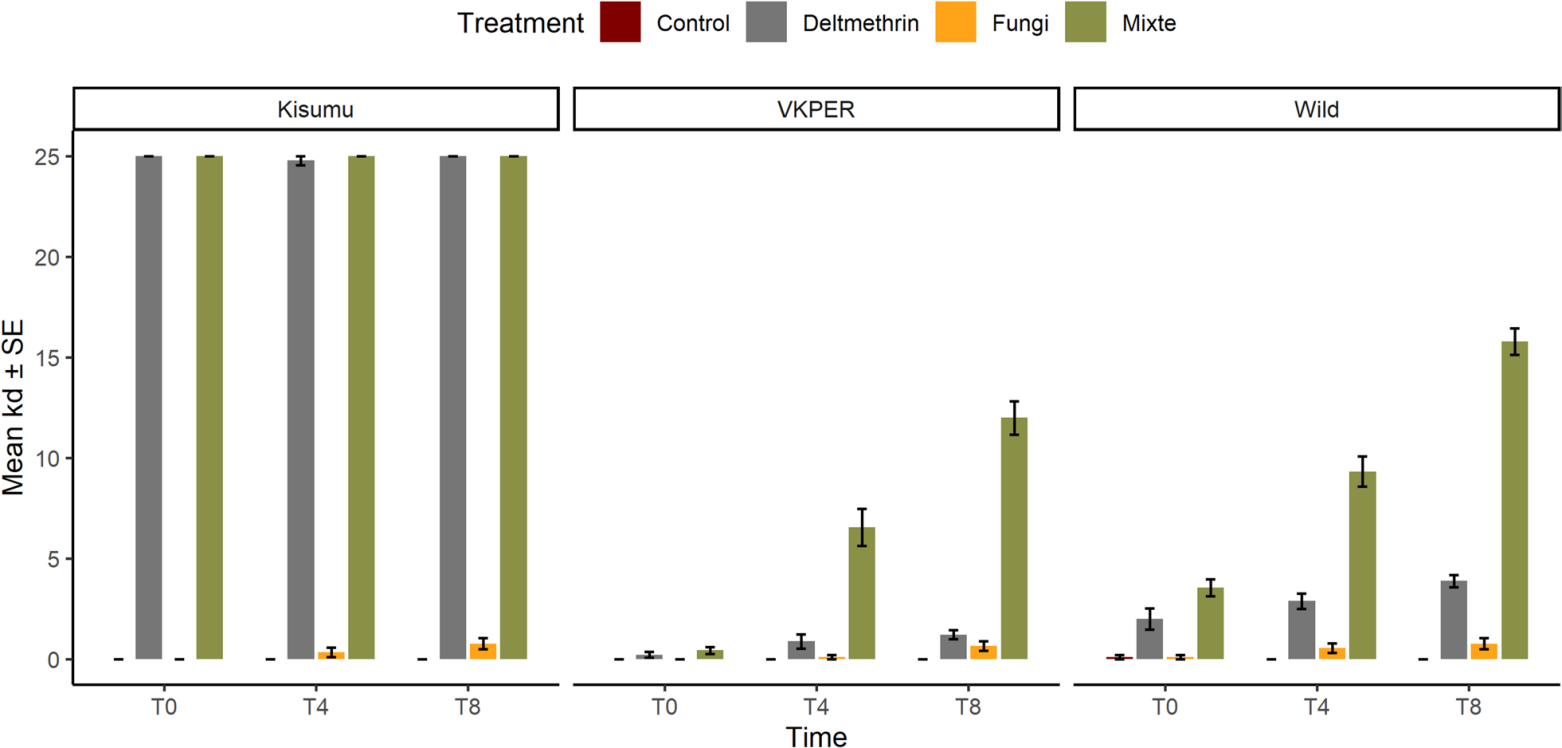
Proportion of dead mosquitoes in the insecticide-susceptible strain *An. gambiae* s.s. Kisumu (left panel) and two insecticide-resistant strains *An. coluzzii* VKPER (middle panel) and wild *An. gambiae* s.l. (right panel). Mosquitoes were exposed to either a Tween solution (Control), *Metarhizium* suspension only (Fungi), deltamethrin 0.05% only (Deltamethrin), a *Metarhizium* suspension, or a combination of *Metarhizium* and deltamethrin (Mix)

To assess the phenotypic effects of *Metarhizium* infection on *Anopheles* mosquito susceptibility to insecticides, we infected *Anopheles coluzzii* VKPER, *An. gambiae* Kisumu and wild *An. gambiae* s.l. with *Metarhizium*. On days 0, 4, and 8 post-infection, the mosquitoes were exposed to deltamethrin for 1 h. Mortality was recorded 1 h after the insecticide exposure. The binomial GLM model showed significant effects of treatment, time, and strain on mosquito mortality (*p* < 0.001 for all the factors). Combination of deltamethrin and *Metarhizium* resulted in the highest mortality rate 27.5% (95% CI 24.9–30.2) followed by deltamethrin 5.4% (95% CI: 4.3–6.6), then *Metarhizium* 1% (95% CI: 0.6–1.6) (Fig. 2). Pairwise comparisons revealed statistically significant differences between treatments (Table 1), confirming the efficacy of the combined treatment compared to the individual applications.

Gene expression has complex associations with mosquito strains, treatment, and infection duration.

#### CYP6P3 expression

**Fig. 3:**
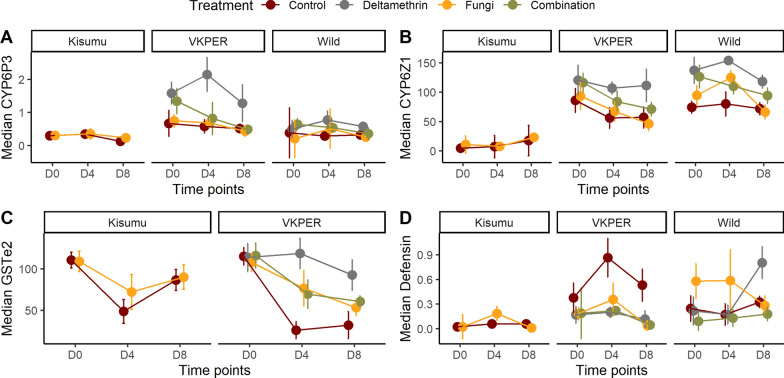
Median expression levels (± SE) of **A** *CYP6P3*, **B** *CYP6Z1*, and **C** *GSTe2* detoxification genes, along with **D** median *defensin,* an immune gene in *Anopheles gambiae* Kisumu (susceptible), *Anopheles coluzzii* VKPER (resistant), and wild *Anopheles* mosquitoes. Mosquitoes were exposed to four treatments: Control (red), Deltamethrin (gray), *Metarhizium* (yellow), and a combination of deltamethrin + *Metarhizium* (green). Measurements were taken at three time points: Day 0 (baseline for gene expression), Day 4, and Day 8 post-treatment

To investigate the role of *CYP6P3* in the reversal of insecticide resistance by *Metarhizium* infection, we analyzed its expression at three time points during the course of *Metarhizium* infection. The best model for *CYP6P3* transcription included mosquito strain (ꭓ^2^ = 18.22, df = 2, p < 0.001), time (ꭓ^2^ = 8.77, df = 2, *p* < 0.01), and treatment (ꭓ^2^ = 12.81, df = 3, *p* < 0.05). *CYP6P3* transcription was significantly upregulated in *An. coluzzii* VKPER by 58.9% (95% CI: 28.3–89.5, *p* < 0.001) compared to *An. gambiae* Kisumu. Although deltamethrin exposure alone increased *CYP6P3* transcription by 52.4% (95% CI: 21.1–83.7, *p* < 0.01), deltamethrin in combination with *Metarhizium* infection reduced *CYP6P3* levels by 36.3% (95% CI: 10.9–61.7, *p* < 0.01) compared to deltamethrin only, suggesting that infection modulates the metabolic response to the insecticide (Fig. 3A).

#### CYP6Z1 expression

To investigate the role of *CYP6Z1* in the reversal of insecticide resistance by *Metarhizium* infection, we analyzed its expression at three time points after *Metarhizium* infection. Mosquito strain (ꭓ^2^ = 53.99, df = 2, *p* < 10^–6^) and treatment (ꭓ^2^ = 19.1, df = 3, *p* = 0.0002) had significant effect on *CYP6Z1* expression level. *CYP6Z1* transcription was respectively 47.7% (95% CI: 30.9–64.5, *p* < 0.001) and 67.3% (95% CI: 50.5–84.1, *p* < 0.001) higher in *An. coluzzii* and wild *An. gambiae* s.l. than in *An. gambiae* Kisumu, suggesting a potential role of this gene in metabolic insecticide resistance.

In mosquitoes exposed to deltamethrin alone, *CYP6Z1* transcription increased by 35.8% (95% CI: 18.6–52.9, *p* < 0.001) compared to *An. gambiae* Kisumu. However, in those previously infected with *Metarhizium*, subsequent deltamethrin exposure increased *CYP6Z1* transcription only by 21.8% (95% CI: 4.4–39.3, *p* < 0.05), highlighting that *Metarhizium* can alter the metabolic response to the insecticide exposure (Fig. 3B).

#### GSTe2 expression

To investigate the role of the *GSTe2* gene in the reversal of insecticide resistance by *Metarhizium* infection, we analyzed its expression at three time points during the course of *Metarhizium* infection. *GSTe2* transcription was impacted by mosquito strain (ꭓ^2^ = 5.10, df = 1, *p* = 0.02) and the interaction between treatment and infection duration (ꭓ^2^ = 12.91, df = 6, *p* = 0.04). The insecticide-resistant *An. coluzzii*, *GSTe2* levels were lower by 17.4% (95% CI: -32.5–2.4, *p* < 0.05) compared to the reference insecticide-susceptible strain *An. gambiae* s.s. Kisumu.

In mosquitoes exposed to deltamethrin only, *GSTe2* transcription was 73.2% (95% CI: 28.0–118.3, *p* = 0.0033) and 34.8% (95% CI: -10.3–79.9, *p* = 0.15) higher on days 4 and day 8, respectively, than their corresponding control groups (control days 4 and control day 8). However, when mosquitoes were infected with *Metarhizium* and exposed to deltamethrin, GSTe2 transcription was only 39.0% (95% CI: -6.2–84.2, *p* = 0.11) and 5.7% (95% CI: -39.5–50.8, *p* = 0.81) higher on days 4 and 8 compared to the within group control day 0, suggesting that *Metarhizium* infection modulates *GSTe2* expression (Fig. 3C).

#### *Defensin* expression

To investigate the role of d*efensin* in the reversal of insecticide resistance by *Metarhizium* infection, we analyzed its expression at three time points during the course of *Metarhizium* infection. The best model for *defensin* transcription included mosquito strain (ꭓ^2^ = 20.92, df = 2, *p* < 0.001) and treatment (ꭓ^2^ = 9.16, df = 3, *p* = 0.02). The *defensin* gene was overexpressed in *An. coluzzii* VKPER, and wild *An. gambiae* s.l. compared to *An. gambiae* Kisumu. The estimates were 35.4% (95% CI: 18.6–52.2, *p* = 0.00006) and 37.9% (95% CI: 21.1–54.8, *p* = 0.000018), respectively. In contrast, *defensin* transcription was reduced in mosquitoes treated with a combination of *Metarhizium* and deltamethrin by 22.7% (95% CI: 5.2–40.2, *p* = 0.0123742) compared to untreated *An. gambiae* Kisumu, similarly to deltamethrin alone (Fig. 3D).

#### Fungal infection depletes energy reserves

To assess the effect of *Metarhizium* infection on mosquito fitness, we quantified key energy levels and reserves, including glucose, glycogen, and lipids. The experiments were conducted using insecticide-resistant *An. coluzzii* VKPER and insecticide-susceptible *An. gambiae* Kisumu at the three time points.

#### Glucose quantification

**Fig. 4:**
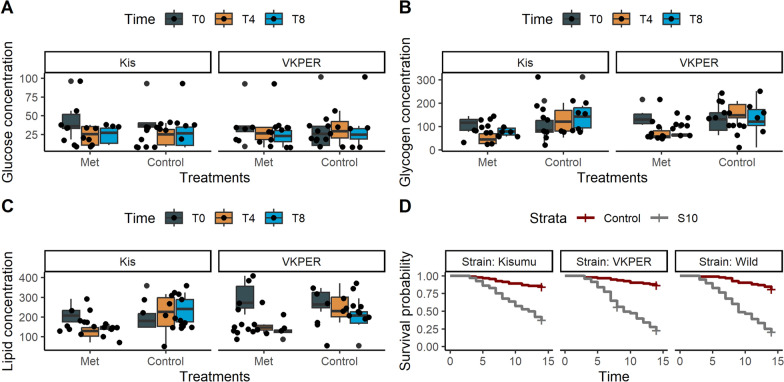
Changes in metabolite concentrations (± SE) in *Anopheles* mosquitoes following *Metarhizium* infection. **A** Glucose, **B** Glycogen, **C** Lipid concentrations were measured in the insecticide-susceptible strain *Anopheles gambiae* Kisumu (left panels) and the insecticide-resistant strain *Anopheles coluzzii* VKPER (right panels) after *Metarhizium* infection, and **D** mosquito survival over 14 d after *Metarhizium* infection. Metabolite levels were assessed at three time points: Day 0 (baseline for metabolite concentration), Day 4, and Day 8 post-treatment. The x-axis represents the treatments, and the y-axis indicates metabolite concentrations (µg/µL). Error bars denote the standard error of the means

Only infection duration (time) was retained as significant in the final model (ꭓ^2^ = 11.98, df = 2, *p* < 0.005). Across both treatments (control and fungi) and mosquito strains (Kisumu and VKPER), a consistent decline in glucose concentration was observed over time. Specifically, glucose concentration decreased by 15.5% (SE = 5.425, *p* = 0.005) 4 days and 17.6% (SE = 5.425, *p* = 0.001) 8 days after *Metarhizium* infection compared to the baseline (day 0). These results indicate that *Metarhizium* infection does not affect glucose concentration, even during infection (Fig. 4A).

#### Glycogen quantification

We found a strong interaction between fungal infection and time on glycogen concentration in mosquitoes (ꭓ^2^ = 11.288, df = 2, *p* = 0.003). In both the susceptible and resistant strains, the mean glycogen levels in infected mosquitoes declined significantly as the infection progressed. In the control group, glycogen levels remained the highest, averaging 118.58 µg/L (95% CI: 90–147, *p* < 0.00001). However, in the infected group, glycogen levels decreased significantly by 77.0% (95% CI: 27.6–126.4, *p* = 0.003) on day 4 post-infection and by 66.5% (95% CI: 17.1–115.9, *p* = 0.01) on day 8. This revealed a progressive depletion of energy reserves in the mosquitoes as the infection duration increased (Fig. 4B).

#### Lipid quantification

The interaction between fungal infection and infection duration was retained as significant in the final model (ꭓ^2^ = 10.318, df = 2, *p* = 0.005). In both the susceptible and resistant strains, the mean lipid levels in infected mosquitoes significantly decreased as the infection progressed. Lipid level in the control group remained the highest, averaging 243.34 µg/L (95% CI: 206.6–280.1, *p* < 0.00001). However, in the infected group, lipid levels decreased significantly by 46.2% (95% CI: 16.1–76.3, *p* = 0.004) on day 4 post-infection and by 38.4% (95% CI: 8.3–68.5, *p* = 0.01) on day 8 (Fig. 4C) compared with baseline day 0.

### Survival

The Hazard Ratio of dying in mosquitoes infected with fungi was 7.503 (95% CI: 5.38–10.45) times higher compared to non-treated control mosquitoes. HR for laboratory insecticide-resistant *An. coluzzii* VKPER and field caught mosquitoes were 1.35 (95% CI: 0.98–1.85) and 1.48 (95% CI: 1.08–2.01), respectively. These results indicated a significant increase in hazard compared to the reference insecticide-susceptible *An. gambiae* ss Kisumu (Fig. 4D).

## Discussion

This study provides new insights into the mechanisms underlying insecticide resistance reversal via fungal infections in mosquitoes. In particular, fungal infection impairs the expression of detoxifying genes and reduces mosquito energy reserves, hinting at a dual mechanism by which *Metarizhium* can re-establish susceptibility, even in highly pyrethroid-resistant mosquitoes.

The results of this study revealed the complex interactions among fungal infections, insecticide exposure, and gene expression in mosquitoes. The upregulation of *CYP6P3* and *CYP6Z1* in *An. coluzzii* VKPER, and wild *An. gambiae* s.l. compared to the insecticide-susceptible *An. gambiae* ss Kisumu confirmed a strong association between these genes and insecticide resistance in malaria mosquitoes [15, 16, 47, 52, 53]. In contrast, GSTe2 transcription was lower in *An. coluzzii* VPER compared with *An. gambiae* s.s. Kisumu. GSTs are typically associated with detoxification of DDT, whereas metabolic resistance to pyrethroids is often associated with cytochrome P450 monooxygenases and esterases [53, 54]. As *An. coluzzii* VKPER was selected for pyrethroid resistance, it might be useful to confirm whether the VKPER strain exhibits cross-resistance to carbamates or whether it has elevated GST activity despite its selection history. Otherwise, a strain specifically selected for carbamate resistance would be more appropriate.

The upregulation of *defensin* transcription in insecticide-resistant *An. coluzzii* VKPER, and wild *An. gambiae* s.l. than in the susceptible *An. gambiae* Kisumu suggested that *defensin* may be involved in the immune response to environmental stressors, including insecticide exposure and fungal infections. *Defensin* is a key antimicrobial peptide that plays an important role in the immune defense of mosquitoes, particularly in response to microbial infections such as bacterial and fungal infections [55].

Interestingly, *Metarhizium* infection led to a decrease in *CYP6P3* transcription, a key gene involved in insecticide detoxification. Fungal infections potentially weaken the detoxification capacity of mosquitoes, as suggested by Lamy et al. [35]. This hypothesis was supported by Reingold et al., who demonstrated that infection with *Metarhizium brunneum* affects gene transcription in aphids [56]. Although deltamethrin treatment induced significant increases in *CYP6P3* and *CYP6Z1* transcription, the combination of *Metarhizium* and deltamethrin had only a moderate effect. This result suggests that *Metharhizium* infection inhibits *CYP6P3* and *CYP6Z1* transcription in mosquitoes, even when they are exposed to deltamethrin.

*GSTe2* expression was higher in response to deltamethrin exposure, but the effect was less pronounced in *Metarhizium* infection. These findings highlight the role of detoxification genes in the insecticide resistance of malaria vectors. These results demonstrate the potential of *Metarhizium* infection to inhibit metabolic insecticide resistance mechanisms. *Metarhizium pingshaense* can therefore act as a synergist, enhancing the efficacy of deltamethrin, and making it a valuable tool for integrating malaria vector control strategies in the current context of widespread insecticide resistance.

This study revealed that fungal infection significantly reduced glycogen and lipid levels in mosquitoes as well as their survival. We showed that glycogen and lipid concentrations in *Metarhizium*-exposed mosquitoes were significantly lower than in control mosquitoes, but this was not the case for glucose concentration, regardless of the mosquito colony. The current results support our hypothesis that *Metarhizium* infection causes energy depletion in mosquitoes [35]. In *Metarhizium anisopliae*, the penetration of the insect cuticle is facilitated by high pressure within the appressoria. This is achieved through the degradation of intracellular lipids into glycerol, which accumulates and increases osmotic pressure [57]. This process may explain the observed reduction in the lipid content of the infected mosquitoes.

These findings align with those of previous studies, demonstrating that *Metarhizium* infection can impair mosquito behavior and fitness. For instance, *Metarhizium*-infected *Aedes aegypti* mosquitoes have been shown to fly shorter distances than their uninfected counterparts, thereby reducing their dispersal capabilities and ability to locate hosts for blood meals [58]. This reinforces the potential of *Metarhizium* not only as a direct pathogen, but also as a disruptor of essential behaviors that contribute to the reduction of mosquito vectorial capacity. No significant variations in glucose concentrations were observed between the treatment groups. Because both groups were given access to feed from a 5% glucose solution, this may explain the lack of significant differences in glucose levels between the groups. Additionally, glucose may originate from the conversion of glycogen and lipids [59].

The concentrations of glucose, glycogen, and lipids decreased over time, with this decrease being more pronounced in infected mosquitoes. On days 4 and 8, glucose, glycogen, and lipid levels were significantly lower than those on day 0. This suggests that as mosquitoes grow older, their energy reserves diminish, affecting their fitness compared with younger individuals, as previously demonstrated by Somé et al. [60].

Survival in *Metarhizium*-infected mosquitoes was significantly lower than that in controls. Previous studies have shown a similar biopesticide potential of *Metarhizium* fungi, including the strain S10 used in this study [28]. Wild *Anopheles gambiae* s.l., presumed to be resistant to insecticides, were found to be more susceptible to *Metarhizium* infection than laboratory-reared, insecticide-susceptible *An. gambiae* s.s. Kisumu strain. No difference was observed in insecticide-resistant *An. coluzzii* VKPER. The reduced survival observed in wild *An. gambiae* s.l*.* mosquitoes during this study is likely due to the combined effects of environmental stressors and field-acquired infections. Unlike laboratory-reared mosquitoes, which are bred under controlled conditions that may affect their immune responses and overall susceptibility to pathogens, such as *Metarhizium*, wild mosquitoes face diverse environmental pressures and pathogen exposure. These external factors may enhance susceptibility profiles and influence survival outcomes [35]. This finding is consistent with that of Howard et al. [29], who demonstrated that insecticide-resistant mosquitoes exhibit increased susceptibility to entomopathogenic fungi compared with insecticide-susceptible strains. Furthermore, in an insecticide-free environment, the *kdr* mutation, which confers resistance to pyrethroids, was found to enhance mosquito survival by reducing neural and behavioral excitability in resistant individuals [61, 62]. However, this is disadvantageous to *Plasmodium falciparum*-infected mosquitoes [61, 63].

## Conclusions

This study highlights a potential mechanism by which the entomopathogenic fungus *Metarhizium* significantly reduces mosquito survival and reverses insecticide resistance, thereby diminishing their disease transmission potential. Beyond reducing survival, *Metarhizium* infection impairs mosquito fitness by depleting key energy reserves, including glycogen and lipids, and by altering metabolic and immune gene expression. Our findings further demonstrate that the longer the infection persists, the greater is the impact on energy reserves, intensifying the physiological effect on the host. These results underscore the multifaceted effects of *Metarhizium* infection on mosquito biology and offer valuable insights into its potential as a biological control agent. *Metarhizium*-based strategies may play a pivotal role in the integrated vector management strategies aimed at reducing malaria transmission.

## Author contributions

LDL, EB, and FB conceived and designed the study. LDL implemented the study. LDL, MV, FB, and EB analyzed the data and wrote the original draft of the manuscript. EB, NM, and DA revised the final manuscript. All the authors have read and approved the final version of the manuscript.

## Funding

This work was supported by the Wellcome Trust grant, Ref 218771/Z, and the National Institute for Health Research (NIHR) (using the UK’s Official Development Assistance (ODA) Funding). LDL is supported by the Ministry of Higher Education Research and Innovation of Burkina Faso and by the Wellcome Trust grant, Ref 218771/Z, and the National Institute for Health Research (NIHR) (using the UK’s Official Development Assistance (ODA) Funding). MV is funded by the European Research Council, European Union’s Horizon 2020 Research and Innovation Programme grant 852957. FB was supported by an Academy of Medical Sciences Springboard Award (ref: SBF007–100094). The proofreading fees for this manuscript were supported by the African Research Initiative for Scientific Excellence (ARISE) grant (ref: ARISE-PP-FA-143) awarded to Dr. Etienne Bilgo. The ARISE programme is funded by the European Union and implemented by the African Academy of Sciences, in partnership with the African Union Commission and the European Commission.

## Data availability

Data supporting the conclusions of this article are included within the article and its additional files.

## Supplementary Information

Supplementary Material 1

Supplementary Material 2

Supplementary Material 3

Supplementary Material 4
